# Subcortical brain volume changes linked to concern of falling in multiple sclerosis: a structural MRI study

**DOI:** 10.1007/s00415-025-13287-7

**Published:** 2025-08-02

**Authors:** Shay Menascu, Tali Drori, Alon Kalron

**Affiliations:** 1 Multiple Sclerosis Center, Sheba Medical Center, 2 Derech Sheba Street, Tel Hashomer, Israel; 2 Department of Physical Therapy, Stanley Steyer School of Health Professions, Gray Faculty of Medical and Health Sciences, Tel-Aviv University, Tel-Aviv, Israel; 3 Sagol School of Neurosciences, Tel-Aviv University, Tel-Aviv, Israel

**Keywords:** Multiple sclerosis, MRI, Free surfer, Concern of falling, Fear of falling

## Abstract

**Introduction:**

Concern of falling (CoF) affects approximately 50–60% of people with MS (pwMS) and is associated with physical and cognitive deficits. Despite its functional impact, CoF’s neuroanatomical correlates in pwMS are poorly understood.

**Objectives:**

Using structural MRI, we investigated associations between subcortical brain volumes and CoF in a large pwMS cohort.

**Methods:**

This study involved 407 pwMS that were divided into three groups based on their CoF, as assessed by the Falls Efficacy Scale-International (FES-I). Volumetric MRI analysis was performed using FreeSurfer to assess subcortical brain structures, including the basal ganglia, hippocampus, and corpus callosum. Analyses were adjusted for age, sex, intracranial volume, scanner type, and magnetic field strength.

**Results:**

Significant differences in brain volume were found between groups with low, moderate, and high CoF. PwMS with high CoF exhibited reduced volumes in the pallidum, putamen, and corpus callosum compared to those with low CoF (p < 0.05). Regression analysis revealed that subcortical volume reductions, particularly in the basal ganglia, were significantly associated with higher CoF scores, even after controlling for disability.

**Conclusions:**

CoF in pwMS is associated with structural brain changes in areas related to motor control and emotional regulation. This may indicate that CoF reflects both psychological and neuroanatomical factors.

## Introduction

Multiple sclerosis (MS) is a chronic inflammatory disease of the central nervous system characterized by progressive neurological disability, commonly including impairments in gait and balance [1, 2]. Consequently, falls are a frequent and clinically significant problem among people with MS (pwMS); over half experience at least one fall within a six-month period [3]. Beyond the immediate risk of injury, falls often lead to psychological consequences, most notably fear or concern of falling (CoF). CoF is a highly prevalent issue in this population, affecting approximately 50–60% of pwMS, and is associated with physical and cognitive deficits [4]. Specifically, poorer performance in cognitive domains such as attention and executive function has been linked to greater levels of CoF [5], suggesting that this phenomenon is multifactorial, reflecting a combination of motor, cognitive, and emotional factors.

Although CoF’s clinical features and functional impact in pwMS have been relatively well described, its underlying neuroanatomical correlates remain poorly understood. Evidence from other populations, such as older adults and individuals with Parkinson’s disease (PD), suggests that specific brain alterations may contribute to the emergence or maintenance of CoF [6, 7]. For example, voxel-based morphometry in older adults has revealed associations between greater CoF and reduced gray matter volume in the superior frontal gyrus, supplementary motor area, and cerebellum, areas involved in motor planning, balance, and cognitive control [6]. In PD, patients experiencing freezing of gait have demonstrated reduced functional connectivity between the amygdala and frontoparietal networks, and the degree of this disconnection correlates with higher CoF [7]. These findings indicate the involvement of circuits integrating emotion regulation, motor control, and executive function in the pathophysiology of CoF.

Despite evidence in other populations, no studies have examined structural brain correlates of CoF, specifically in pwMS. Therefore, it remains unclear whether CoF in MS reflects only a psychological reaction to previous falls and/or balance difficulties or stems from brain structure changes.

For several reasons, identifying the structural neural correlates of CoF in pwMS is scientifically important. First, it may enhance the early identification of patients at risk of developing severe CoF or subsequent falls. If specific neuroimaging markers, such as regional atrophy, can be linked to CoF, they could serve as accessible tools for risk stratification using standard clinical MRI. Second, it would advance our understanding of how psychological (e.g., fear) and physical aspects (e.g., balance difficulties) interact in MS. CoF often triggers a cascade of activity avoidance, physical deconditioning, social withdrawal, and depression, all of which contribute to accelerated functional decline [8]. Finally, investigating how CoF relates to brain structure may help clarify whether it reflects a broader disease burden, such as MS-related neurodegeneration, or whether it involves specific, potentially modifiable brain changes that could be targeted through interventions like physical training, cognitive-behavioral therapy, or neuromodulation.

Therefore, this study aimed to compare volumetric measures of subcortical brain structures in pwMS according to their CoF. Importantly, we present subcortical brain volume scores relative to normative data while controlling for potential confounding variables, including age, sex, total intracranial volume, MRI field strength, and scanner manufacturer, which are known to affect regional brain segmentation [9]. We hypothesized that pwMS with greater CoF would exhibit reduced volumes in basal ganglia structures and the hippocampus compared to those with little or no CoF.

## Methods

### Study design and participants

Our retrospective cross‐sectional study comprised 407 pwMS (41.2 ± 13.1 years, 66.6% female) recruited from the Multiple Sclerosis Center, Sheba Medical Center, Tel‐Hashomer, Israel. Data were extracted from the center’s computerized database, a population‐based registry documenting demographic, clinical, and imaging data of all consecutive pwMS followed at the center. The integrity of the data registry was evaluated by a computerized logic‐algorithm‐questioning process identifying data entry errors. A computerized questionnaire was used to assist in choosing pwMS according to the following inclusion criteria: (i) a neurologist‐confirmed diagnosis of definite MS according to the revised McDonald criteria [10]; (ii) an Expanded Disability Status Scale (EDSS) ≤ 6.5, equivalent to walking ~ 20 m with bilateral support [11]; (iii) a brain MRI performed using the three‐dimensional high‐resolution fast spoiled gradient‐echo MS protocol; (iv) completion of the Falls Efficacy Scale-International (FES-I) [12]; and (v) a brain MRI and concern of falling status assessed within a 6‐month period. Exclusion criteria included (i) corticosteroid treatment within 60 days prior to the brain MRI and/or completion of the FES-I; (ii) pregnancy; (iii) other significant neurological or psychiatric illnesses; (iv) started or stopped disease-modifying treatment within 90 days of brain MRI and/or completion of the FES-I; and (vii) participation in a clinical trial involving an active rehabilitation program within 90 days prior to completion of the FES-I and brain MRI. An anonymous code number referenced each patient’s record to ensure confidentiality during the statistical analyses. The study was approved by the Sheba Institutional Review Board Ethics Committee (Ethics ref. 5596‐08/141210) in addition to a full exemption from written or verbal consent from the study participants. Hence, individual data will be unavailable to protect the participants’ identity.

### MRI acquisition protocol

All patients completed a whole-brain MRI performed by a high-resolution 8-channel head coil 3-Telsa MR General Electric Signa scanner. The imaging sequence used was a 3-D fast spoiled gradient-echo protocol with an isotropic voxel size of 1 × 1 mm, echo time (TE) = 2 ms, repetition time (TR) = 6 ms, inversion time (TI) = 450 ms, 146 contiguous sagittal slices with a field of view of 256 X 256 mm, matrix 256 × 256 mm and a flip angle of 20. The MRI protocol was identical for all patients.

### Post-acquisition MRI data processing

Volumetric analysis was based on the three‐dimensional T1‐weighted images by the FreeSurfer image analysis suite (version 5.1; http://surfer.nmr.mgh.harvard.edu/) on a 64‐bit Linux CentOS 5. The automated procedures for subcortical volumetric measures of the different brain structures have been described [13, 14]. Procedures for measuring the subcortical volume have been validated against histological analysis [15] and manual measurements [16, 17]. Furthermore, the FreeSurfer morphometric procedures have exhibited good test–retest reliability by scanner manufacturers and field strengths [18]. Subcortical regions of interest (ROI) in the present study were determined by previous studies investigating balance difficulties, falls, and/or fear on brain volume in adults [19–21]. The subcortical ROI in this hypothesis‐driven analysis were volumes (mm^3^) of the hippocampus, amygdala, brain stem, thalamus, accumbens nucleus, corpus callosum, and the basal ganglia (putamen, caudate, pallidum) regions. The volume of each subcortical ROI (produced by the FreeSurfer software) was computed by the freely available “Subcortical Norms Calculator” obtained from the Alzheimer’s Disease Neuroimaging Initiative [22]. A detailed description of the Microsoft Excel spreadsheet was provided by Potvin et al. analysis [23]. In brief, the spreadsheet computed the estimate of the expected subcortical regional volumes of the individual based on age, sex, estimated total cranial volume, scanner manufacturer, and the scanner magnetic field. Data used to produce normative values were obtained via an anatomical MRI performed on 2790 healthy individuals aged 18–94, using 23 samples provided by 21 independent research groups [24]. The spreadsheet offered a normative reference against which the subcortical volume of clinical populations was compared. The spreadsheet was computed separately for each MS individual. In each case, we extracted the estimated percentile of the subcortical ROI according to the adjusted normative population, i.e., a score of 23% for the thalamus indicated that the pwMS thalamic volume was estimated to be in the 23rd percentile of the thalamic volume of healthy adults, matched for age, gender, total cranial volume, type of MRI scanner and magnetic field.

### Concern of falling (CoF)

The participant’s self-reported questionnaire, the Falls Efficacy Scale International (FES-I), a common measure of CoF [12], assesses the level of concern regarding falling during 16 activities of daily living ranging from basic to more demanding, including social activities that may contribute to the quality of life. For each item, the level of concern is scored on a four-point scale (1 = not concerned at all, 4 = very concerned) within a total range of 16–64 points. The higher the score, the more concerned the subject is about falling. The FES-I has shown excellent internal and test–retest reliability (Cronbach’s alpha = 0.96, ICC = 0.96) and construct validity in different populations, including pwMS, and has been suggested for use in cross-cultural rehabilitation research and clinical trials [25]. In the present study, we divided the study sample into three concern groups with respective cut-off points: low concern: 16–19; moderate concern: 20–27; and high concern: 28–64, based on Delbaer et al. validation study [26].

### Statistical analysis

The sample was divided into three groups according to their CoF: low, moderate, and highly concerned. Descriptive statistics determined the participants’ demographics, clinical characteristics, and subcortical brain measures according to their group allocation. Group differences in age and gender distribution were determined by the independent sample t-test and chi‐squared test, respectively. Analysis of variance tests with multiple corrections determined the differences in disability, subcortical volume, and the estimated percentile point in terms of adjusted norms between subgroups.

A hierarchical logistic regression analysis, comprising two blocks, was performed; the FES-I score was defined as the dependent variable. The first block of the regression analysis (employing the forward conditional method) included the estimated percentile point of the subcortical ROI inserted as independent variables. Disability, represented by the EDSS, was added to the independent variable list in the second block. All analyses were performed using SPSS software (version 29.0 for Windows; SPSS Inc., Chicago, IL, USA). All reported p-values were two‐tailed. The level of significance was set at p < 0.05.

## Results

The clinical characteristics of the 407 participants are summarized in Table 1. The mean FES-I scores for the low, moderate, and high concern groups were 16.9 (SD = 1.1), 22.8 (SD = 2.4), and 40.3 (SD = 9.0), respectively. PwMS classified as highly concerned were older, had a longer disease duration, and exhibited greater disability, as indicated by higher EDSS scores, compared to those in the low and moderate concern groups.

**Table 1:** Characteristics of the study sample

Variable	Total (n = 407)	Low concern (n = 141)	Moderate concern (n = 103)	High concern (n = 163)	p-value
Age (years)	41.2 (13.1)	36.2 (12.4)	41.3 (13.4)	45.5 (11.9)	< 0.001
Gender (F/M)	271/136	75/66	73/30	124/39	< 0.001
Disease duration (years)	6.6 (8.6)	3.4 (5.8)	5.7 (7.4)	9.8 (10.0)	< 0.001
MS type (RR/P)	360/47	132/9	90/13	138/25	0.218
EDSS (score)	2.6 (1.8)	1.2 (1.1)	2.3 (1.4)	3.9 (1.5)	< 0.001
Pyramidal	1.4 (1.2)	0.6 (0.8)	1.4 (1.2)	2.2 (1.1)	< 0.001
Cerebellar	0.9 (1.0)	0.3 (0.6)	0.8 (1.0)	1.4 (1.1)	< 0.001
Sensory	0.9 (1.0)	0.4 (0.8)	0.9 (1.0)	1.3 (1.1)	< 0.001
FES-I (score)	27.8 (12.0)	16.9 (1.1)	22.8 (2.4)	40.3 (9.0)	< 0.001

**Fig. 1:**
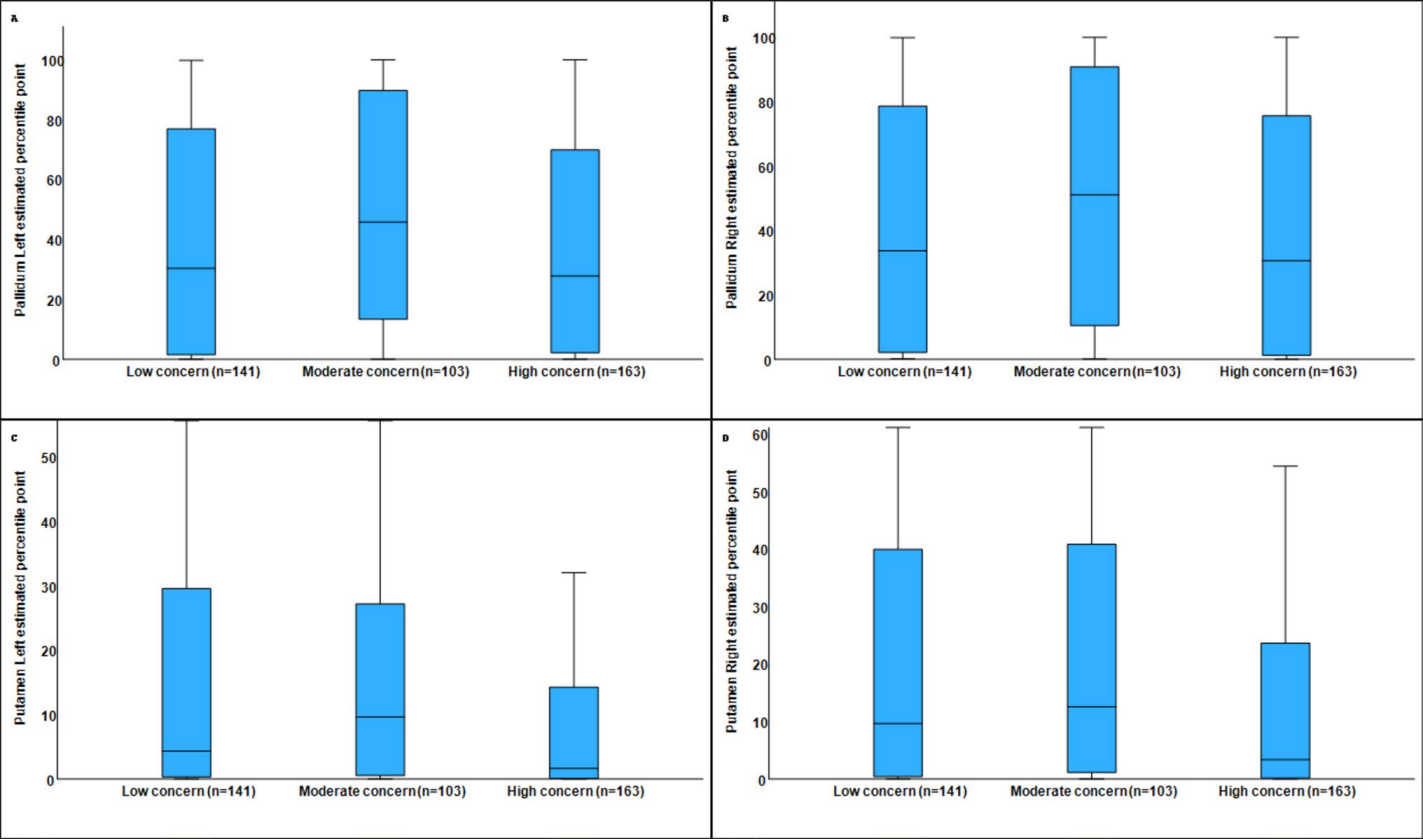
Estimated scores for the left pallidum (**A**), right pallidum (**B**), left putamen (**C**), and right putamen (**D**) by concern-of-falling group

Table 2 presents the estimated percentile scores of subcortical ROIs according to group allocation. Significant differences were identified in the pallidum, putamen, corpus callosum, and brainstem. Compared to the low and high concern groups, the highest estimated percentile in the pallidum (i.e., closest to the normative reference) was observed in the moderate concern group. The lowest estimated percentile in the putamen was found in the high concern group, compared to the low and moderate concern groups. Additionally, pwMS in the high-concern group demonstrated lower estimated percentiles in the brainstem and corpus callosum relative to the other two groups. Figure 1 illustrates the estimated scores for the putamen and pallidum with regard to the concern of falling groups. No differences between CoF subgroups were found regarding the accumbens, amygdala, caudate, hippocampus, thalamus, and total subcortical brain volume.

**Table 2:** Estimated percentile point of brain ROI based on the normative population, adjusted for age, gender, magnetic field strength, MRI manufacture, and intracranial volume

Brain ROI	Total (n = 407)	Low concern (n = 141)	Moderate concern (n = 103)	High concern (n = 163)	p-value/effect size
Accumbens Lt	37.0 (31.2)	37.6 (31.6)	41.1 (31.1)	33.9 (30.8)	0.179/0.031
Accumbens Rt	32.2 (28.6)	34.6 (29.7)	35.5 (29.0)	32.2 (28.6)	0.626/0.016
Amygdala Lt	30.5 (29.8)	33.7 (31.3)	30.5 (28.8)	27.7 (28.9)	0.221/0.029
Amygdala Rt	36.3 (32.7)	39.0 (34.0)	37.6 (31.7)	33.2 (32.2)	0.276/0.027
Caudate Lt	26.0 (27.5)	28.4 (29.6)	24.0 (23.5)	25.1 (28.1)	0.401/0.023
Caudate Rt	24.8 (26.1)	25.7 (26.8)	26.0 (25.8)	23.2 (25.8)	0.600/0.017
Hippocampus Lt	46.2 (38.3)	48.7 (39.3)	47.2 (35.3)	43.4 (39.2)	0.468/0.021
Hippocampus Rt	38.3 (33.8)	41.7 (35.0)	37.4 (30.9)	35.9 (34.6)	0.317, 0.025
Pallidum Lt	41.7 (37.2)	40.1 (37.0)	49.4 (37.1)	38.3 (37.0)	**0.049/**0.043
Pallidum Rt	42.7 (37.0)	39.3 (36.6)	50.6 (37.7)	40.5 (36.3)	**0.039/**0.045
Putamen Lt	15.6 (23.0)	18.5 (25.2)	17.2 (21.6)	12.2 (21.6)	**0.044/**0.044
Putamen Rt	19.9 (25.3)	22.6 (26.2)	23.3 (25.7)	15.3 (23.7)	**0.012/**0.055
Thalamus Lt	30.5 (31.2)	29.3 (30.5)	32.2 (30.9)	30.4 (32.2)	0.778/0.012
Thalamus Rt	23.1 (28.2)	23.1 (25.8)	26.4 (30.7)	21.0 (28.5)	0.319/0.025
Corpus callosum	5.7 (17.0)	8.9 (22.4)	5.5 (15.4)	3.3 (11.8)	**0.036/**0.056
Brain stem	27.7 (28.9)	29.3 (29.3)	32.5 (28.5)	23.3 (28.2)	**0.028/**0.048
Subcortical gray volume	6.3 (19.1)	5.2 (19.2)	6.5 (19.6)	7.4 (18.6)	0.608/0.017

Bold indicates *p*-value <0.05

Table 3 presents a hierarchical logistic regression analysis using the estimated percentile scores of subcortical regions. A reduced score in the right putamen was significantly associated with higher FES-I scores, indicating greater CoF. Including brainstem scores in the model increased the explained variance, while the association with the right putamen remained statistically significant.

**Table 3:** Logistic regression analysis to examine the relationship between the FES-I score (dependent variable) and the estimated percentile point of the brain ROI according to adjusted norms (independent variable)

Model	Variable	β	95% CI	p-value	*R* square
1	Constant	29.883	28.112, 31.654	< 0.001	0.030
	Putamen Rt	−0.080	−0.130, −0.031	0.002	
2	Constant	31.260	29.102, 33.418	< 0.001	0.044
	Putamen Rt	−0.065	−0.116, −0.014	0.013	
	Brain stem	−0.052	-0.098, −0.005	0.030	

## Discussion

Our primary observation was that CoF is associated with specific subcortical brain structures. To the best of our knowledge, this is the first study to investigate the neural correlates of concern of falling in individuals with MS. Notably, this study used the estimated percentiles of subcortical volumes relative to an age- and sex-adjusted normative population as the primary brain measure since we believe that this brain metric is more informative than traditional absolute volume measurements.

The study’s key finding is the association of the pallidum and putamen with levels of CoF. Specifically, pwMS with high concern have a relatively smaller putamen volume (adjusted to norms) compared to those with low or moderate concern, who did not differ from each other. In contrast, pallidum volume did not differ between individuals with high and low concern; however, both groups had relatively smaller volumes than those with moderate concern, whose volumes were closest to the normative value. No significant differences were observed between concern of falling subgroups in other subcortical regions, such as the thalamus, hippocampus, or amygdala, emphasizing the specific involvement of the putamen and pallidum in relation to CoF.

The putamen and pallidum, both key components of the basal ganglia, play essential roles in motor control, postural regulation, and the integration of sensorimotor information, functions that are directly relevant to balance and fall risk [27, 28]. The putamen is particularly involved in the planning and execution of voluntary movement and in refining motor output, making it critical for maintaining a stable gait and posture [27]. Structural alterations in this region may impair the smooth and coordinated execution of motor commands, potentially increasing instability and elevating CoF. The pallidum, particularly its internal segment, contributes to motor regulation through its influence on thalamocortical pathways and plays a vital role in controlling postural tone and movement inhibition [28].

Beyond their motor functions, the putamen and pallidum are recognized for their involvement in fear-related behaviors. Based on several mechanistic studies, these structures act as an interface between emotional and motor systems, facilitating the translation of fear into adaptive motor responses [29, 30]. Notably, ventral pallidum neurons are necessary to generalize and express fear-related responses in minimal threat contexts [31]. Activation of these regions during cognitively driven fear has been shown to engage cortical-striatal-thalamic loops, which are involved in threat evaluation and executing context-appropriate behavioral responses [32]. In the case of concern of falling, such neural activity may reflect a state of persistent motor vigilance or caution, potentially manifesting as altered gait patterns, reduced confidence, and compensatory behaviors in individuals at risk, such as pwMS. Together, these findings support the notion that structural and functional changes in the putamen and pallidum may contribute to objective balance impairments and subjective CoF in pwMS, highlighting the dual motor-emotional role of these subcortical regions.

In addition to the basal ganglia, our results also indicated significantly lower brainstem volumes in the high CoF group compared to the low and moderate concern groups. The brainstem plays a central role in postural control, vestibular processing, and the coordination of automatic motor responses, functions that are directly relevant to balance maintenance and fall prevention [27]. Structural compromise in this region may impair integration of visual, proprioceptive, and vestibular inputs necessary for stable gait and reactive balance. Although the brainstem did not emerge as a primary independent predictor in our regression model, its inclusion increased the explained variance, suggesting a contributory role alongside basal ganglia structures. This finding supports the idea that CoF in pwMS may reflect broader dysfunction across multiple sensorimotor regulatory systems.

Interestingly, in several subcortical structures, such as the pallidum and brain stem, the moderate concern subgroup showed the most normative percentile values, indicating brain structure measures closest to normative levels, compared to the low and high concern groups. A similar, though non-significant, trend was observed in the thalamus and nucleus accumbens. These findings suggest that moderate CoF may reflect an adaptive response, enhancing vigilance and promoting safer behavior, unlike low or high concern, which may reflect under- or overestimating fall risk. On the contrary, a moderate degree of concern may serve as an adaptive response that promotes vigilance and risk awareness. This has been shown in several adult populations, particularly elders [33, 34]. Accordingly, it is plausible that pwMS who report moderate CoF may display more normative structural brain characteristics in specific regions relative to those with high concern, who may impose unnecessary restrictions on their activities, or those with low concern, who may underestimate their fall risk and engage in unsafe behaviors. Future research is warranted to clarify this issue further, preferably via longitudinal studies.

Our study has many strengths, such as the relatively large cohort; however, several limitations warrant attention. First, as a cross-sectional study, causal relationships between brain structure and CoF cannot be inferred. Secondly, although our analysis focused on subcortical gray matter volumes using FreeSurfer, we did not include other MRI modalities, such as diffusion tensor imaging, functional MRI, MR spectroscopy, or magnetization transfer imaging, which could have offered valuable complementary insights into microstructural and functional alterations, particularly within white matter tracts involved in motor and balance control. Most importantly, we did not quantify white matter lesion burden, a core pathological feature of MS that is strongly associated with both motor and cognitive dysfunction. Including lesion load metrics would have enabled a more comprehensive assessment of the relative contributions of gray matter atrophy and white matter damage to CoF. Future research should aim to integrate both gray and white matter measures, ideally using multimodal imaging approaches, to more fully elucidate the neural underpinnings of CoF in pwMS. Also, CoF was assessed using a single self-reported questionnaire (FES-I), which, although well-validated and widely used in MS populations, captures only one aspect of this complex, multifactorial construct. Additional instruments, such as the Activities Balance Confidence scale [35] or the Fear of Falling Avoidance Behavior Questionnaire [36], might have provided complementary insights, but all self-report measures are subject to inherent limitations. We also did not include direct assessments of psychological or cognitive factors such as fatigue, depression, anxiety, or executive function, which are known to correlate with CoF [37]. However, these variables are deeply interrelated in MS and often overlap in their associations with both behavior and brain structure. Attempting to control for each separately in a cross-sectional design presents methodological challenges, including risks of multicollinearity and overadjustment. Extensive prior research has already demonstrated associations between mood, cognition, and brain structure in MS [38, 39]. In contrast, our study aimed specifically to examine whether CoF itself is linked to neuroanatomical differences. Nonetheless, future studies should incorporate longitudinal designs and broader behavioral profiling to better disentangle these overlapping influences.

## Conclusion

This study provides novel evidence linking elevated CoF in pwMS to reduced volumes in specific subcortical brain regions, particularly within the basal ganglia. These findings suggest that CoF in pwMS may not merely reflect a psychological reaction to prior falls or balance difficulties but may also be associated with structural brain changes. This study highlights the potential relevance of subcortical brain alterations as objective markers of fall-related concern by utilizing normative volumetric reference data and adjusting for key demographic and scanner-related variables. These insights deepen our understanding of the neural mechanisms behind CoF in MS and highlight the need to integrate both behavioral and neurobiological assessments into clinical practice.

## Data Availability

Data are available upon reasonable request.
